# Editorial: Pathological livers in the surgery of hepatic resections and liver transplantation, volume II

**DOI:** 10.3389/fmed.2023.1330122

**Published:** 2023-11-17

**Authors:** Marc Micó-Carnero, Andrés Trostchansky, Carmen Peralta

**Affiliations:** ^1^Department of Liver, Digestive System and Metabolism, Institut d'Investigacions Biomèdiques August Pi i Sunyer, Barcelona, Spain; ^2^Universitat de Barcelona, Barcelona, Spain; ^3^Departamento de Bioquímica and Centro de Investigaciones Biomédicas (CEINBIO), Facultad de Medicina, Universidad de la República, Montevideo, Uruguay

**Keywords:** liver transplantation, ischemia reperfusion (I/R) injury, partial hepatectomy, steatosis, liver surgery

Deceased organ donation consists of both donation after circulatory death (CD) and donation after brain death (BD) (1). The majority of liver transplants (~80%) involve organs from BD donors, with 4–20% originating from CD donors (2). Both CD and BD result in significant hemodynamic alterations, mesenteric microcirculation hypoperfusion, and warm hepatic ischemia, culminating in inflammation and cell damage, ultimately leading to deleterious effects on the liver grafts used for transplantation (3, 4).

To reduce transplant waiting lists, the use of marginal liver grafts could expand the pool of available organs by using deceased donors. Marginal livers can be classified into two different categories: those with a higher risk of impaired function (e.g., those from older patients, people who are steatotic, diabetic donors, and split livers, among others), and grafts carrying the risk of infection or malignancy for the recipient (e.g., donors with viral infections or cancer) (5). This topic is of clinical and scientific interest since there will soon be an increased need for liver grafts from marginal donors to alleviate transplant waiting lists and because of the prevalence of metabolic disorders in liver resections.

There is an urgent demand for therapeutic, surgical, and technological strategies to alleviate the detrimental effects of BD and CD on liver grafts, improve the tolerance of marginal livers to ischemia/reperfusion (I/R) injury, and address regenerative failure in partial hepatectomies and liver transplants (6). Despite numerous studies, the influence of each variable related to marginal donors on graft function, recipient survival, and post-surgical outcomes in major liver surgeries remains under investigation due to controversial results (7). Furthermore, the molecular mechanisms underlying the harmful effects of BD and CD on transplanted liver grafts are being studied and more studies have to be performed to report new signal pathways involved in this process (2, 8, 9). Understanding the common molecular signaling pathways that underlie various liver pathologies, including BD and CD, as well as their role in hepatic I/R, is crucial for clinical practice. Discussions and studies in this field are essential for developing effective interventions to enhance postoperative outcomes for marginal livers undergoing transplantation or resection and deceased organ donation. These advances would not only alleviate transplant waiting lists by enabling the use of grafts from extended criteria donors but also lead to improved outcomes for pathological livers undergoing resection, thereby enhancing the clinical outlook for liver surgery in the future.

There is also an unmet need for swift and non-invasive methods to assess the degree of steatosis and the presence of liver pathologies in donors, as these factors significantly impact transplantation prognosis. The establishment of novel approaches in liver transplantation and hepatic resections requires such tools.

This Research Topic encompasses a range of contributions, including basic, translational, and clinical research, original studies, reviews, systematic reviews, brief research reports, case reports, and mini-reviews, focusing on the mentioned areas.

The articles published on this topic show advances in the field of major liver surgeries as partial hepatectomies or liver transplantation, which could be of scientific and clinical interest. The first investigation presented in this issue is a case report that indicates the efficacy of associating liver partition and portal vein ligation for staged hepatectomy (ALPPS) in patients with hepatocarcinoma (HCC) who underwent immunotherapy previously for the first time, which may be an alternative salvage option for HCC therapy (Ning et al.). Other results shown in this issue identify and summarize the existing evidence regarding enhanced recovery after surgery (ERAS) failure and related risk factors after hepatic surgery. This systematic review and meta-analysis concluded that the frequently identified risk factors for ERAS failure after hepatic surgery are linked to operative and anesthesia factors, with major resection being a crucial aspect. Nevertheless, the authors highlight the necessity of performing more randomized controlled trials with standardized evaluation frameworks for ERAS programs (Ren et al.). On the other hand, a review presented on the current topic discusses and shows the new advances made to detect the early signs of sterile inflammation and the extent of this damage as well as potential therapeutic options. This process named sterile inflammation is of clinical relevance in the context of liver surgery because it is the immune response associated with DAMPS released during cell death (Kahan et al.). Finally, the last manuscript in the collection elucidates on the prevalence of systemic circulating tumor cells (CTCs) before and after the resection of HCC and detects differences in CTCs between open liver resection (OPEN) and laparoscopic liver resection (LAP) cohorts. In comparison to conventional OPEN technology, LAP technology can augment the quantity of epithelial, mixed, and mesenchymal circulating tumor cells (CTCs). The study concluded that there was an increase in the total number of CTCs within the LAP group (Lei et al.). This special topic consists of different manuscripts reporting new data about potential clinical applications in the field of major liver surgeries. The editors believe that they are of clinical and scientific relevance and further research in this direction is crucial for improving the clinical outcomes of liver transplantation and partial hepatectomy.

## Author contributions

MM-C: Writing—original draft. AT: Writing—review & editing. CP: Writing—review & editing.
